# How to Distinguish Non-Inflammatory from Inflammatory Pain in RA?

**DOI:** 10.1007/s11926-024-01159-4

**Published:** 2024-08-09

**Authors:** Sharmila Khot, George Tackley, Ernest Choy

**Affiliations:** 1 Department of Anaesthesia, Intensive Care and Pain Medicine, Cardiff and Vale University Health Board, Cardiff CF14 4XW and Cardiff University Brain Research Imaging Centre (CUBRIC), Cardiff University, Maindy Road, Cardiff, Wales CF24 4HQ UK; 2 Cardiff University Brain Research Imaging Centre (CUBRIC), Cardiff University, Maindy Road, Cardiff, Wales CF24 4HQ UK; 3 Head of Rheumatology and Translational Research at the Division of Infection and Immunity and Director of the Cardiff Regional Experimental Arthritis Treatment and Evaluation (CREATE) Centre at Cardiff University School of Medicine, Cardiff, Wales, UK CF14 4YS

**Keywords:** Rheumatoid arthritis, Nociplastic, Inflammatory, Chronic pain

## Abstract

**Purpose of the Review:**

Managing non-inflammatory pain in rheumatoid arthritis (RA) can be a huge burden for the rheumatologist. Pain that persists despite optimal RA treatment is extremely challenging for patient and physician alike. Here, we outline the latest research relevant to distinguishing non-inflammatory from inflammatory RA pain and review the current understanding of its neurobiology and management.

**Recent Findings:**

Nociplastic pain is a recently introduced term by the international pain community. Its definition encompasses the non-inflammatory pain of RA and describes pain that is not driven by inflamed joints or compromised nerves, but that is instead driven by a functional reorganisation of the central nervous system (CNS).

**Summary:**

Insights from all areas of nociplastic pain research, including fibromyalgia, support a personalised pain management approach for non-inflammatory pain of RA, with evidence-based guidelines favouring use of non-pharmacological interventions. Future developments include novel CNS targeting pharmacotherapeutic approaches to treat nociplastic pain.

## Introduction

Rheumatoid arthritis is a chronic, inflammatory, autoimmune disease that mainly affects joints but is known to have extra articular effects on pulmonary, nervous and cardiovascular systems [1]. Pain remains a widely prevalent symptom in RA despite substantially improved disease modifying antirheumatic drugs (DMARDs). Indeed, pain is often disproportionate to disease activity measures and frequently persists even where clinical, biochemical and imaging evidence of inflammation has resolved [2, 3].

The focus of rheumatological management tends to be disease control and the biomedical model has historically viewed pain as a symptom secondary to disease, with the in-built assumption that treatment of the disease should eliminate pain. Therefore, the patient who has ongoing pain complaints may suspect their symptoms are being dismissed as less important than disease control or even feel ‘not believed’ [4].

Helpfully, there is a growing recognition that inflammatory and non-inflammatory pain phenotypes can co-exist in RA [5–9]. Herein we use 'inflammatory pain' to describe pain that is proportionate to the level of RA activity, as measured by inflammatory markers and joint imaging, and 'non-inflammatory pain' to describe pain disproportionate to RA presentation. We explore the mechanisms of inflammatory and non-inflammatory pain in RA, recommend some clinical techniques to help differentiate them, and suggest a management approach that can be adapted to tackle multiple underlying pain aetiologies.

## What is Pain?

Pain has been defined as “an unpleasant sensory and emotional experience associated with, or resembling that associated with, actual or potential tissue damage” (International Association for the Study of Pain (IASP) [10, 11]. Pain is always a personal, multidimensional experience, learnt conceptually through life experiences and influenced to varying degrees by neurobiological, psychological, and social factors [11], features that hold true for RA pain [7]. Nociception is the neurobiological process by which a painful stimulus is conveyed by the nervous system to the brain. Pain (the aversive experience) and nociception (the transmission of noxious information) are therefore distinct phenomena: pain cannot be inferred solely from activity in nociceptive neurons.

### Nociception

Nociceptors are sensory receptors that detect molecules or signals from tissues often due to damage and/or inflammation. Nociceptors are at the nerve endings of thinly myelinated (Aδ) and unmyelinated (c) fibres which are the main neuronal fibres implicated in the detection of mechanical, chemical (including inflammatory mediators), and thermal noxious stimuli. These fibres synapse with neurons in the superficial laminae of the spinal cord dorsal horn, a site that is subject to local and distant modulatory inputs (including direct top-down inputs from the brainstem), before ascending predominantly via the spinothalamic tract [12].

The spinothalamic pathway terminates in discrete subdivisions of thalamic nuclei known as the ventral posterior lateral nucleus and the ventromedial nucleus. From these nuclei, nociceptive information is relayed to various cortical and subcortical regions, including the amygdala, hypothalamus, periaqueductal grey (PAG), basal ganglia, and regions of cerebral cortex such as the somatosensory cortices I and II (SI–II), the prefrontal cortex (PFC), the anterior cingulate cortex (ACC) and the insular cortex (IC). Most notably, the insula and anterior cingulate cortex are consistently activated when nociceptors are stimulated by noxious stimuli, and activation in these brain regions is associated with the subjective experience of pain [13]. In turn, these integrated thalamocortical and corticolimbic structures, collectively have been termed by some the pain “neuromatrix,” [14].

**Fig. 1:**
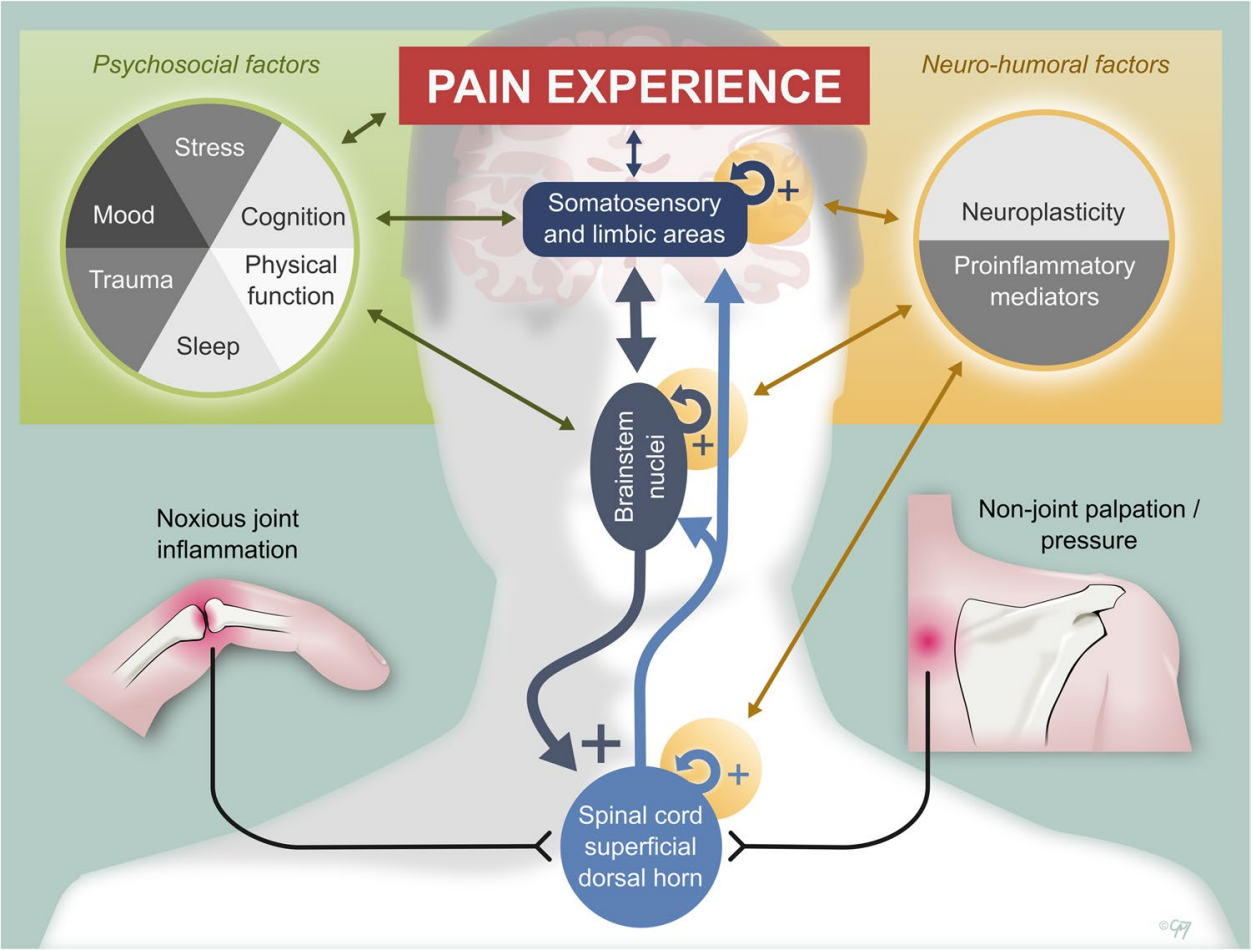
Mechanisms of non-inflammatory pain in RA

Top-down modulation of pain is driven from many brainstem sites, including the PAG, rostroventromedial medulla (RVM) and locus coeruleus (LC), and is largely subserved by noradrenergic and serotonergic neurons. Top-down modulation can both inhibit and facilitate nociceptive transmission at the level of the spinal cord superficial dorsal horn (SDH). The balance between excitatory and inhibitory signals to the secondary sensory neurons helps filter the sensory information reaching the brain [15]. (See Fig. 1)

### Pain in RA

Pain is important for health and survival. It has evolved as a powerful alarm signal that motivates protective behaviours, e.g., nursing a damaged limb, or withdrawing from a damaging stimulus. Pain becomes a problem when it is present but no longer serves this protective role whilst retaining its strongly salient and aversive nature. This pathological pain may be classified as nociceptive, neuropathic or nociplastic [7, 9].

Nociceptive pain is pain caused by actual or threatened damage to tissues and due to the activation of nociceptors [10, 11]. Active synovitis of RA would be an example of nociceptive pain caused directly by inflammation. Neuropathic pain is caused by a lesion or disease of the somatosensory system [10]. This type of pain is often described as burning, shooting, tingling, stabbing and in RA can be caused by nerve entrapment or secondary peripheral neuropathy, e.g. carpal tunnel syndrome, or neuropathic pain secondary to co-existing diabetes.

Nociplastic pain is a new mechanistic descriptor that seeks to capture pain that is neither nociceptive nor neuropathic. This is defined by IASP as "pain that arises from altered nociception despite no clear evidence of actual or threatened tissue damage causing the activation of peripheral nociceptors or evidence for disease or lesion of the somatosensory system causing the pain" [10, 11, 16]. Patients may have a combination of nociceptive and nociplastic pain (10). Nociplastic pain can involve various organ systems e.g. IBS and occur either as a primary pain pathology e.g. fibromyalgia or in tandem with pre-existing nociceptive e.g. in RA or neuropathic pain conditions e.g. in Multiple Sclerosis. Fibromyalgia is considered the archetypal nociplastic pain condition, wherein pain exists without evidence of nociceptive or neuropathic mechanisms. Nociplastic symptoms are common in RA, sometimes fulfilling the fibromyalgia diagnostic criteria (then termed secondary fibromyalgia), and by definition presenting as pain that is out of proportion to underlying RA disease activity [17, 18].

In reality, these three pain pathologies often co-exist. It is perhaps equally helpful then to consider the various mechanisms by which pain might become pathological.

### Mixed Pain

Mixed pain is a complex overlap of the different known pain types (nociceptive, neuropathic, nociplastic) in any combination, acting simultaneously and/or concurrently to cause pain in the same body area. Either mechanism may be more clinically predominant at any point of time. Mixed pain can be acute or chronic [19]. However, some mixed pain may also be a manifestation of an entirely independent pathophysiological mechanism, and this is an area that needs further research [20].

### Inflammatory Pain in RA

The treatment of inflammatory pain is a staple of rheumatological practice. It is largely understood as a nociceptive form of pain, originating from synovial and periosteal nociceptors signalling inflammatory changes [21].

### Mechanisms of Inflammatory Pain in RA

Pain is a cardinal feature of inflammation. Inflammatory joint pain in RA is a type of nociceptive pain initiated by pro-inflammatory mediators such as prostaglandins, bradykinins and neurotrophic growth factors released during synovial inflammation [22]. These proinflammatory mediators induce an inflammatory cascade and the synoviocytes interact with cells of the adaptive and innate immune system further causing a hyperplastic synovium, bone erosions and cartilage destruction [21]. Nociceptors innervating the synovium and subchondral bone are responsible for arthritic pain; these include joint nociceptors specialised in the detection of chemical stimuli, including the above inflammatory mediators, as well as mechanical or thermal noxious stimuli [23]. In RA chronic inflammation is also thought to cause structural and functional alterations in the peripheral innervation of joints leading to pain [24].

Cytokines and chemokines such as CXCL1 act on peripheral terminals of nociceptor neurons activating them and causing pain [23, 25]. Inflammation sensitises nociceptors to noxious and innocuous stimulation reducing firing thresholds. Noxious inflammatory mediators such as cytokines and prostaglandin E2 released by innate immune cells have been shown to stimulate pain and pain sensitisation in the carrageenan mouse model of inflammatory pain [26, 27] and the Complete Freund’s Adjuvant (CFA) arthritis model [23].

### Clinical Clues and Manifestations of Inflammatory Pain

Inflammatory pain tends to be worse after inactivity so typically early morning. It is also associated with stiffness. Joint swelling and tenderness are the signs and symptoms of synovial membrane inflammation following immune activation and can be elicited via clinical examination and imaging (e.g. joint ultrasound). Elevations of the erythrocyte sedimentation rate (ESR) and C-reactive protein (CRP) level are consistent with the presence of an inflammatory state and can reflect the degree and extent of (local) synovitis and systemic inflammation in RA. Joint swelling and raised inflammatory markers are therefore commonly combined to assess disease activity, such as in the 28-joint Disease Activity Score (DAS28) [28]. Unsurprisingly, raised CRP levels are also associated with greater pain in RA [29]. Interestingly, systemic inflammation and autoimmunity are detectable several years prior to the onset of detectable joint inflammation [30] and autoantibody positivity and pain is highly predictive for development of RA [31], suggesting pain symptoms may precede diagnosis of RA, and highlighting the nuanced dichotomy of inflammatory and non-inflammatory pain mentioned above.

### Non-Inflammatory Pain of RA

Several diseases with inflammatory and immune mediated pathogenesis such as inflammatory bowel disease [32], multiple sclerosis [33] and RA [3] include reports of pain severity being disproportionate to disease activity. Indeed, a third of patients diagnosed with RA report significant and severe widespread pain out of proportion to measures of systemic inflammation [3]. This out-of-proportion pain is often labelled 'non-inflammatory'.

Other causes of non-inflammatory pain in RA not covered in this article, include mechanical damage to articular surfaces causing nociceptive pain [34] and compressive neuropathies leading to neuropathic symptoms [35, 36].

Non-inflammatory pain in RA may sometimes manifest before evidence of inflammation and commonly persists despite adequate control of inflammation. The former suggests that mechanisms other than inflammation can contribute to nociplastic pain [37], findings that are corroborated in animal models [38, 39].

Non-inflammatory pain in RA rarely presents in isolation but instead is usually found alongside inflammatory pain aetiologies in something of a continuum. There is evidence that non-inflammatory and inflammatory pain share common aetiologies during the earlier stages of the disease, with pro-inflammatory pathways causing hyper-nociception in early RA and providing an environment for the potential development of nociplastic pain [23, 40].

### Mechanisms of Non-Inflammatory Pain of RA

See Fig. 1 for an overview of non-inflammatory pain mechanisms. Non-inflammatory pain in rheumatoid arthritis (RA) is maintained via altered pain processing in the central nervous system (CNS). This manifests as pain out of proportion to underlying RA disease activity, including pain amplification from joint inflammation and from typically innocuous stimuli (e.g. pressure delivered to non-joint regions, such as the medial border of the scapula).

Pain is amplified in the brain and superficial dorsal horn of the spinal cord (SDH). The SDH is the site of integration of ascending pain signals (primary sensory neurons), spinal interneurons, and descending modulatory inputs. The SDH is thus subject to top-down (descending modulatory) influences that facilitate pain transmission and is vulnerable to neuroplastic changes that may arise as a consequence of persistent pain (or inflammatory) states. Local inflammatory milieu, including activated microglia, cytokines (such as IL6) and chemokines, can also have direct effects facilitating pain transmission.

Brainstem nuclei (such as the rostro-ventromedial medulla, the locus coeruleus, peri-aqueductal grey, amongst many others) receive bottom-up pain input from the SDH, and top-down input from several brain regions, including those related to attention and executive function (e.g. dlPFC & ACC), mood and fear (e.g. amygdala), context and memories (e.g. hippocampus). The top down and bottom-up inputs integrate in the brainstem contributing to the descending modulatory influences that act directly on the SDH (primarily via serotonergic and noradrenergic neurons). These cortical areas involved in pain processing are also vulnerable to neuroplastic change and the inflammatory milieu and have a complex relationship with the individual's psychological state and their social environment (See [9, 12, 13, 15] for review).

### Central Sensitisation

Central sensitisation (CS) is defined as an amplification of neural signalling within the CNS that elicits pain hypersensitivity [16] either in the form of allodynia (perceiving innocuous stimuli as painful) or hyperalgesia (perceiving exaggerated pain in response to painful stimuli) [41]. Allodynia and hyperalgesia are typical features of neuropathic and nociplastic pain conditions. CS is sometimes referred to as ‘pain augmentation’.

CS cannot be directly determined in humans but certain tools are available, although primarily used in research, that can provide some clues to its presence in RA. Lowered pressure pain thresholds in non-joint regions, high scores on neuropathic pain symptoms scales (such as Pain Detect Questionnaire, PDQ) and central sensitisation measures (Central Sensitisation Inventory, CSI), high tender joint count (TJC) and patient global assessment of disease activity (PtGA) have all been shown to associate with dysregulated central pain processing in RA [42–53].

### Temporal Summation and Wind-up

Repeated painful stimuli ordinarily lead to increased pain perception of the same stimulus. This phenomenon is termed temporal summation. Temporal summation is a feature of all healthy pain systems, however enhanced temporal summation, which may arise secondary to uncontrolled joint inflammation in RA, is considered a marker of 'pathological' CNS change [54]. Repetitive and high-intensity stimulation due to peripheral joint inflammation causes increased responsiveness of primary and secondary sensory neurons by up-regulating excitatory receptors in the spinal cord dorsal horn [55]. If left uninterrupted this has the potential for reorganisation of synaptic connections in the nervous system resulting in neuroplasticity and chronic nociplastic pain in RA patients [56].

### Altered Descending Modulatory Control

Descending modulation is the mechanism by which brain processes have influences on pain processing at the level of the spinal cord. These top-down mechanisms can facilitate or inhibit pain transmission [15].

The periaqueductal grey (PAG), rostroventromedial medulla (RVM), nucleus raphe magnus (NRM) and locus coeruleus are some of the key brainstem regions recognised to be involved in descending modulatory processes, exerting their influences via descending noradrenergic and serotonergic efferents synapsing in the SDH [57–61].

Dysregulation of these pathways can either lead to disinhibition or facilitation, both causing pain amplification [18, 44]. Serotoninergic noradrenergic reuptake inhibitors (SNRIs) may exert some of their analgesic actions here [62, 63].

Conditioned pain modulation (CPM) uses a “pain inhibits pain” principle that is theorised to reveal the strength of descending inhibition [64]. It uses a painful conditioning stimulus to influence (in health: to *inhibit*) a painful test stimulus [65, 66]. Within RA, in-line with the non-inflammatory pain hypothesis, inefficient CPM is predictive of a reduced response to DMARD’s [44]. In these patients then, a different approach to pain management would be needed.

### Role of Cytokines with Special Focus on IL6

Major cytokines involved in RA that have been shown to have direct effect on sensory neurones in experimental systems include TNF, IL-6, IL-1, IL-17. IL-6 is a proinflammatory cytokine that contributes to pathogenesis of RA and circulating IL6 levels are higher in RA patients than healthy controls. Preclinical research supports the hypothesis that IL-6 contributes to RA-associated symptoms and co-morbidities through its effects on the HPA axis [67]. IL-6 has a key role in nociplastic pain, supposedly through activation of the Jak/STAT3 pathway, chemokine overexpression and glial-cell activation [68–71]. Modulation of the IL-6 pathway may therefore attenuate pain-like behaviour in nociplastic pain in RA [67, 68, 72].

### Role of Microglia

Microglia are macrophage-like immune cells found in the CNS. Chronic activation of microglia causes secretion of pro-nociceptive mediators including proinflammatory cytokines such as TNF and IL-1β, chemokines, reactive oxygen species (ROS), and proteases which have all been implicated in the development of central sensitisation [73–75]. Macrophages play a crucial role in RA cytokine-induced inflammation [76].

Microglia's influence on synaptic plasticity and contribution to brain inflammation [77] are considered so critical to chronic pain that some researchers describe chronic pain as a gliopathy [75, 78]. Peripheral inflammation in RA is thought to be essential for activation of microglia and activated M1 microglia directly induce pain via proinflammatory mediators such as IL-1β, TNF, IL-6 causing synaptic plasticity and hypernociception in animal models of RA [40, 76]. Thus, microglia are considered prime future targets for developing CNS-modulating analgesic treatments for nociplastic RA pain [79].

### Role of Toll like Receptors (TLRs) and Chemokines

Toll-like receptors (TLRs) are a family of receptors that are involved in the recognition of conserved pathogen-associated molecular patterns, highly expressed in synovial tissues of RA patients [80]. Functional, genetic or pharmacological blockade of TLRs reverses arthritis in different experimental models [81]. Of relevance to nociplastic pain, MyD88, an adaptive protein recruited by TLRs, is thought to play an important signalling role in arthritic pain by stimulating proinflammatory cytokine release, in turn promoting production of prostaglandins and sympathetic amines. Murine studies have shown that TLR/MyD88 signalling is required in the hypernociceptive response seen after chemically induced joint inflammation [82, 83]. Early preclinical studies report a role for TLR4 antagonists in reversal of inflammation in RA and improvement in allodynia and hyperalgesia in nerve pain models and therefore of potential benefit for both inflammatory and nociplastic pain in RA [84].

Autoantibodies against citrullinated protein (ACPA) are found in ~50% of RA patients [85]. In mice injected with human and murinised ACPA, long lasting prolonged pain like behaviour, in the absence of inflammation, has been demonstrated. This pronociceptive effect was coupled with release of nociceptive chemokine CXCL1 which is an analogue of human IL-8 [86] and a candidate cytokine implicated in maintaining pain states [87]. IL-8 inhibitors have been shown to inhibit neuropathic pain [88] and may potentially benefit RA pain [46, 87].

### Clinical Clues and Manifestations

Nociplastic pain mechanisms are often obscure, however risk factors for nociplastic pain are well described: family history, past pain experience, and psycho-social factors including psychological, emotional and physical trauma, are all recognised to increase the risk of developing nociplastic pain. An 'initiating' risk-factor may be considered a 'trigger', and include stressors that might be psychosocial, or as is often the case for secondary FM in RA, underlying inflammatory disease [89]. Pain severity disproportionate to RA disease activity, disabling fatigue, sleep disturbances or unrefreshed sleep, mood disturbances/disorders, neuropathic symptoms, brain fog, worsening physical and mental health, are all clues indicative of nociplastic pain [18, 90].

Fibromyalgia is the archetypal nociplastic pain and is common in RA patients with a 13-40% prevalence [48, 91–95]. Fibromyalgia has clear diagnostic guidelines, and historically labelled ‘secondary’ fibromyalgia in RA patients can be considered a form of nociplastic pain [17, 89]. A tender joint count (TJC) score 7 or more than the corresponding swollen joint count (SJC) (i.e. TJC minus SJC ≥7) or SJC/TJC ratio of <0.5 is proposed to be highly predictive of concomitant fibromyalgia [48, 94] and therefore of nociplastic pain in RA [18].

Composite disease activity indices commonly used in routine clinical practice to guide ‘treat-to-target’ include Tender Joint Count (TJC) and patient global assessment (PtGA) [96, 97]. Both TJC and PtGA are influenced by the presence of nociplastic pain, leading to higher reported disease activity scores driven by pain unresponsive to escalating RA treatments [49, 53, 98, 99].

Higher reported pain scores are seen in patients with RA reporting disturbed sleep [100], anxiety and depression [101–103] which further contributes to enhanced pain sensitivity, indirectly impacting PtGA and disease activity scores and patient satisfaction with RA treatment.

Pain predicts functioning, health and QOL in RA and is thought to be a more important cause of disability than joint damage [104–106].

Therefore, use of multidimensional questionnaires assessing pain, mood, functioning and sleep may be a useful addition in clinic to assist with mechanistic classification of pain and support appropriate pain management leading to improved patient reported global satisfaction scores [7, 107, 108].

## Management

Maintaining a high index of suspicion for presence of non-inflammatory pain in early RA is key to instituting an early biopsychosocial approach to patient assessment and promoting appropriately combined pharmacological and non-pharmacological therapeutic modalities early in the course of their disease [89, 108]. The NICE Chronic Pain Guidelines visual summary is helpful here (https://www.nice.org.uk/guidance/ng193/resources/visual-summary-pdf-9073473517) [109].

### Developing a Biopsychosocial Perspective

We have emphasised above that pain is not simply a linear track from noxious stimulus to perception but that frequently for RA, pain severity and extent are disproportionate to underlying evidence of active rheumatological disease [110]. Despite maximal treatment of the 'biological' component, with DMARDS, NSAIDs and corticosteroids, pain persists.

Having optimised biological management of RA, and in the presence of clues pointing to a non-inflammatory (nociplastic) aetiology, a careful combination of pharmaceutical and psychosocial targeted therapies should be sought, often with emphasis on the latter. Simple evaluation of psychosocial risk factors in clinic, where appropriate tools are available, can facilitate the institution of early and appropriate treatments, and consideration of referral to chronic pain management services or condition specific self-management programs is recommended [53].

NICE guidelines [109] provide a broad general framework to assess and manage chronic primary nociplastic pain (e.g. fibromyalgia) and chronic secondary pain (e.g. non-inflammatory pain of RA) with an emphasis on shared decision making and a non-pharmacological psychosocial driven approach.

NICE explicitly recommend against the use of standard analgesia for 'chronic primary pain' or pain out-of-proportion with underlying disease (i.e. nociplastic pain, such as fibromyalgia). Instead, NICE recommends using antidepressants with established analgesic profiles for which there is mounting evidence of efficacy within non-nociceptive pain [62, 109, 111].

First-line antidepressants are amitriptyline and duloxetine, favoured for their dual actions on noradrenergic and serotonergic systems [63, 109, 112]. Amitriptyline is particularly beneficial in people with severe sleep disturbances. Tramadol and pregabalin are also options that are recommended for severe pain in fibromyalgia (nociplastic pain) in the European League Against Rheumatism (EULAR) guidelines which predate the NICE 2021 guidance for pharmacological management of chronic primary pain (fibromyalgia) [63, 109].

International EULAR guidelines align with NICE [113] in recommending individualised tailored therapy and emphasising the first-line role of non-pharmacological interventions [63, 89, 114]. Physical activity, exercise interventions, psychological interventions, educational interventions, orthotics, weight management, and multidisciplinary treatment overall have positive effects on pain and are ubiquitously recommended for nociplastic pain [63, 109, 114].

Exercise has a strong evidence base in inflammatory and non-inflammatory pain [115, 116]. Patient preferences should be considered with low impact exercise sometimes proving more sustainable for painful RA [117].

Management should be aimed at improving health-related quality of life and not just pain severity reduction [63, 114]. Psychological therapies are recommended for mood disorder, or in those with unhelpful coping strategies, with CBT deemed effective at producing modest, long-term reductions in pain, disability and improving mood [114].

Cognitive behavioural therapy (CBT) was found to have positive impacts on relieving anxiety, depression, and fatigue in RA. However, effect sizes for relief of pain in RA were small for CBT, with a recent Cochrane review suggesting small reductions in pain and distress [118, 119].

Mindfulness is the awareness that emerges from paying attention to things as they are, on purpose, in the present moment and non-judgementally [120]. Mindfulness practice can reduce pain intensity and unpleasantness [121, 122] and help with depressive symptoms in RA [123–125].

CBT and mindfulness-based therapies for chronic pain have the additional benefit of improving physical functioning. Therefore, mindfulness-based therapies could be offered additionally to CBT for improving pain severity and reducing pain interference and psychological distress in in patients with RA [124, 126].

Multimodal rehabilitation programmes are recommended for those with severe disability.

Other therapies such as TENS machine or massage may provide pain reduction via stimulation of large sensory nerves (conveying innocuous sensations from the skin) suppressing transmission in small pain fibres. However, evidence for such therapies in RA is lacking.

Whilst some therapies such as physiotherapy, exercise and mindfulness would benefit both inflammatory and non-inflammatory pain, we would recommend that analgesic therapy is best guided via identification of pain type.

### DMARD’s and Biologics in RA Pain Management

Some contemporary biologics have recently shown promising analgesic effects on residual or disproportionate pain in RA [127, 128]. IL-6 inhibitors, e.g. tocilizumab and sarilumab, and anti-TNF adalimumab, are known to have analgesic efficacy with emerging evidence that they confer a dual benefit in RA patients, treating both inflammatory disease and non-inflammatory (nociplastic) pain [128, 129]. Similarly, Jakstat-inhibitor Tofacitinib demonstrated analgesic benefit beyond that associated with reduction of inflammation implying an additional reduction of RA non-inflammatory (nociplastic) pain [127].

## Conclusion

Pain in RA is complex and multifactorial but should not be confined to the remit of pain specialists. Pain reduction is an important outcome for patients and non-inflammatory (nociplastic) RA pain benefits from early rheumatological management. Empowered with the latest pain neuroscience knowledge, rheumatologists can support early patient education and navigate a logical approach to pharmacological and non-pharmacological pain management. The apparent disconnect between treat-to-target guidance and patient expectations can then be partially bridged using a biopsychosocial approach to clinical assessment early on in disease presentation. It is known that psychosocial approaches based on the individual presentation and preference often improve quality of life and perceived pain [114, 130, 131]. However, distinguishing pain as a symptom or a disease in its own right can often be difficult and an interdisciplinary approach for difficult to treat RA pain is encouraged and is likely to demonstrate improved clinical outcomes [132].

## Data Availability

No datasets were generated or analysed during the current study.
